# Ecological pollution and health risk monitoring assessment of polycyclic aromatic hydrocarbons and heavy metals in surface water, southeastern Nigeria

**DOI:** 10.5620/eaht.2023007

**Published:** 2023-04-11

**Authors:** Chisom Theresa Umeh, John Kanayochukwu Nduka, Daniel Omeodisemi Omokpariola, Joy Ebele Morah, Ebuka Chidiebere Mmaduakor, Nkechi Helen Okoye, Ekene-Echerebo Ifeoma Lilian, Ifeanyi Favor Kalu

**Affiliations:** Pure and Industrial Chemistry Department, Nnamdi Azikiwe University, Anambra State, Nigeria

**Keywords:** surface water, polycyclic aromatic hydrocarbons, heavy metals, ecological risk, cancer risk

## Abstract

Polycyclic aromatic hydrocarbons (PAHs) and heavy metals (HMs) are predominant pollutants linked with anthropogenic activities across a host of environmental mediums. The level of pollution, ecological and health risk were assessed in surface water from Ekulu in Enugu metropolis, Nigeria for 17 PAHs and selected HMs (As, Cd, Cr, Cu, Pb, Ni, Zn) components. PAHs and HMs were determined using a gas chromatography-flame ionization detector (GC-FID) and atomic adsorption spectrophotometer (AAS). The total PAHs in station A (3.17mg/l), B (1.51mg/l), and C (1.83mg/l) were due to high molecular weight (HMW) PAHs than low molecular weight (HMW) PAHs. HMs contents were within USEPA and WHO minimum contamination levels (MCL) except Cr and Pb. The molecular diagnostics of PAHs showed that incomplete combustion of carbonaceous compounds was dominant, while petrogenic was insignificant across all samples. The ecological indices of PAHs and HMs varied from medium to high pollution due to anthropogenic activities that pose a threat to the ecosystem. The non-carcinogenic models showed that hazard index (HI) ranged from PAHs (0.027 – 0.083) and HMs (0.0067 – 0.087) which is less than unity implying no adverse health issues. The lifetime cancer risk (LCR) for PAHs (4.21×10^–4^ – 9.61×10^–4^) and HMs (1.72×10^–5^ – 3.98×10^–5^) suggested significant cancer risk is possible over some time for a population of 1 in 10,000 and 100,000 for both PAHs and HMs exposure for 70 years. Therefore, there is an urgent need for proper pollution control and mitigation plan to preserve both age groups from being continuously exposed to anthropogenic activities in the Ekulu River and further study should be carried out to monitor the available toxicants.

## Introduction

Steady growth in industrialization and urbanization has reduced the water quality in an aquatic ecosystem, so there is a need for incessant monitoring and protection for the health of humans that utilize the water resources. Natural and anthropogenic processes can introduce heavy metals and polycyclic aromatic hydrocarbons (PAHs) into the water environment. The contribution of natural activities such as rock weathering, wildfires, and volcanic eruption [1][2] to pollutants pollution are insignificant in comparison to anthropogenic sources. Some PAHs and heavy metals are byproducts of anthropogenic activities such as incomplete combustion of some natural materials, urban runoff, wastewater discharge from households and industries, oil spillage, and vehicle and industrial exhaust emissions [3-6]. Heavy metals and PAHs are present in different environmental media including water, soil, sediments, and microorganisms [7-12].

Heavy metals and PAHs are venomous to the environment due to their level of toxicity to public health and biota. They can easily bio-accumulate and bioavailable to aquatic life which finds their way to dietary sources that affect humans through food chain transfer [13-14]. Greater quantities of hazardous chemicals especially HMs and PAHs are deposited in the surface water, middle layer, bottom layer, and sediments as a result of fast anthropogenic expansion accumulating to a certain stage and inflicting toxicity to the ecosystem [15-17]. Rivers act as a sink for these contaminants thereby increasing the ecological risk of the water column. During the translocation of HMs and PAHs in surface water, they undergo various transformations such as precipitation, dissolution, adsorption, and complexation mechanism [3][18-21] which determine their nature of bioavailability [22-23]. Hence, continual environmental issues are envisaged with a possible threat to global human health. Dissemination of uncontrollable quantities of toxic pollutants should be apprehended from contaminating sources to reduce the level of health hazards for easy monitoring of water pollution.

Estimation of pollution status and monitoring assessment of heavy metals and polycyclic aromatic hydrocarbons in the accessible surface water is of benefit to the human and aquatic environment. The background level of the pollutants should be established from various polluting sources to know the existing benchmark for certain changes that might arise in near future. Given that, several studies on monitoring heavy metals and PAHs exposure in surface water, sediments, and groundwater in the river from different global locations have been documented [3][14][20][24-30]. However, limited similar studies have been carried out in surface water from Ekulu at the Enugu metropolis. Ugochukwu et al. [31] and Ugochukwu et al. [32] only studied heavy metals and PAHs on sediments from Enugu. No reports have been given on pollution status and public health assessment of heavy metals and PAHs in cumulative surface water from River Ekulu flowing across production industries in Enugu city which is about 1.5 – 3 kilometers away from the river. According to fact-findings from site visitation and reports from Chime and Ogbuanu, [33] and Ifediegwu et al. [34], Ekulu River being the longest and largest river in the Enugu metropolis is the major source of water supply to average and low-income residents of Iva Valley, UgwuAbor, Ugbo Odogwu Abakpa Nike, and Emene settlements, and also supports industrial, recreational and domestic activities. The coal fields (Iva valley, Onyeama, and Ribadu mines) are situated at the upper course of the Ekulu River with an inflow of acid mine drainage from these abandoned coal fields whereas, in the lower course, the river is utilized by Abakpa and Emene populace for refuse dumping, agricultural, industrial, domestic and swimming [35-36]. Effluent runoffs from paint, abandoned coal mines, allied, pharmaceutical, automobile, and food processing industries sited within the locations of the river bank loaded with various forms of pollutants are leached into the water body [31], thus calls for serious concern. The inhabitants within the River settlements also use the river as a dumping medium for waste and domestic effluents. Improper management of these discharged wastes affects the surface water quality of the Ekulu River. Public dwellers are at risk of HMs and PAHs exposure. Therefore, this present study focused on the assessment of 17 PAHs and heavy metals in the surface water of Ekulu River flowing across industrial settlement, likely sources of these environmental pollutants, ecological status, and possible level of health risk to humans.

## Materials and Methods

### Study area

**Figure 1.**
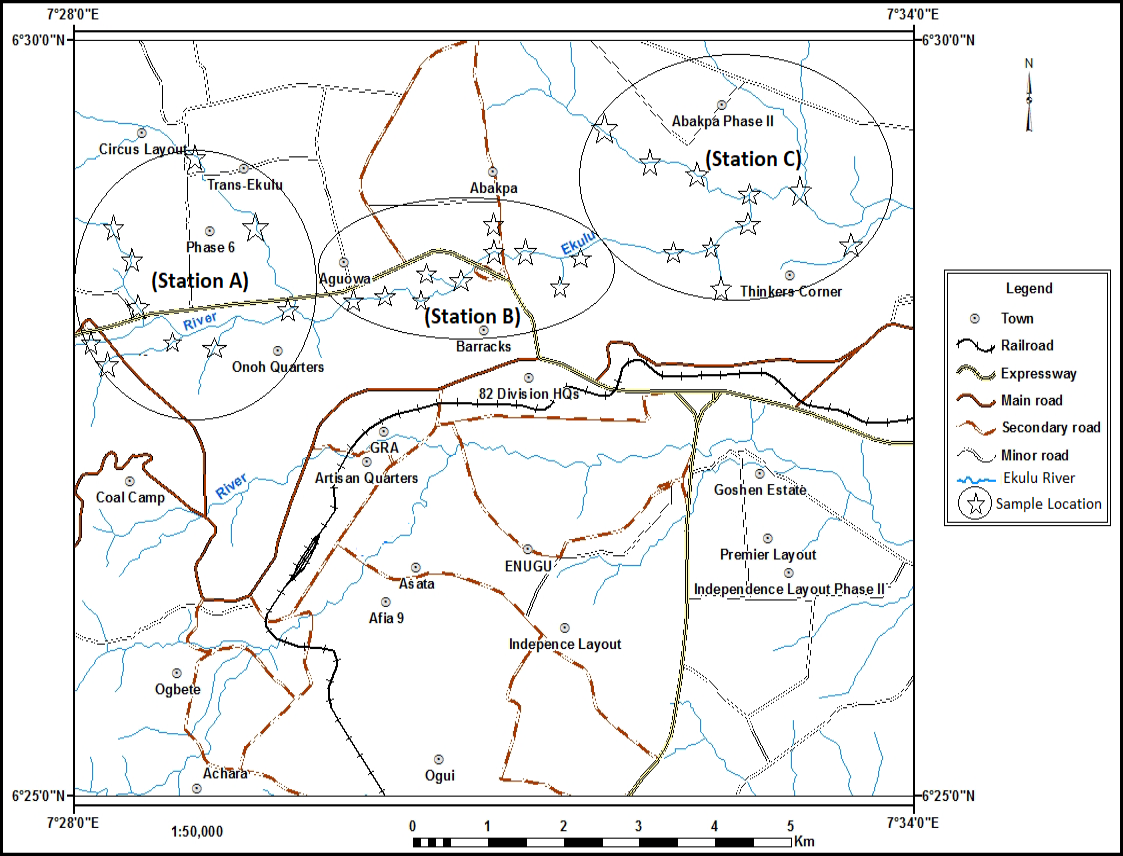
Geological map of the study location

The study area mapped out in Figure 1 indicates sampled locations. It is geographically located in the south-east region of Nigeria with a length of 30.1 km from its source to its confluence with Nyaba River (6°22′00′′–6°30′00′′ N; 7°26′00′′–7°38′00′′ E) having an average basin area of 24.71 km^2^ [34]. It originates from the base of North–South trending Enugu-Okigwe Cuesta in the northern region of Enugu at height of 550 m above sea level, and flow eastward through three local government of the state (Enugu North, South and East). Its major tributaries are Asata, Mkpume-Olu, Nyo, Otuku, Ngene-Aba, Ugwuonu, and Obuga Rivers. The river is mainly used for agricultural (allied), industrial (coal mine, pharmaceutical, automobile, and food processing industries) and other anthropogenic activities. The study location is a derived savannah with temperature ranging from 25°C to 35°C and yearly rainfall of about 950 mm. The study area experiences rainy season and dry season. Ekulu River flows through the city and accumulates discharged wastes from abandoned coal mine and different anthropogenic sources thus affecting the water quality. The nearby inhabitants of the study area used the water for swimming and other domestic purposes.

### Sampling and sample preparations

Thirty surface water subsamples were collected randomly from the river during period of October to December, 2021 designated stations A, B and C comprising of upstream, middle and downstream. Ten water subsamples in each station were homogenized to form composite water sample labeled A. Two other mixed random samples were also collected 5 kilometers away from the first sampled station labeled B and C. The water samples for heavy metal analysis were collected into 1 liter nitric acid pre-washed polyethylene bottles. The water samples were further acidified with 5ml of 6M HNO3, labeled and preserve in a sampled container filled with ice cubes (refrigerator) at temperature of 4oC to prevent the content from microbial growth and then transferred to the laboratory within 24 hours.

For sampling analysis for PAHs in water, the glass bottles were soaked in a 10% HNO3 before they were washed with soap and rinsed successively with tap water, double-distilled water and acetone. Glass wares were subsequently drained and dried in an oven at 105oC for about 12 h. A PAH mix containing naphthalene, acenaphthylene, acenaphthene, fluorene, phenanthrene, anthracene, fluoranthene, pyrene, benzo[a]anthracene, chrysene, benzo[b]fluoranthene, benzo[k]fluoranthene, benzo[a]pyrene, indeno[1,2,3-c,d]pyrene, dibenzo[a,h]anthracene, and benzo[g,h,i] perylene at 2000.0 mg L^−1^ in methanol: methylene chloride (1:1) purchased from Supelco (Bellefonte, USA) was used as standard.

### Extraction of PAHs from samples

The extraction of PAHs from water samples were carried out on a separating funnel following a common procedure for spiked duplicates of PAHs. The 250 cm^3^ separating funnel contains 100 cm^3^ of an equal mixture of chromatographic grade dichloromethane (DCM) and n-hexane. The samples were extracted for 2 hours under reflux; the crude extracts were concentrated to a volume of nearly 2 cm^3^ using a rotary vacuum evaporator. The concentrates were purified by short-column silica gel chromatography using DCM as the eluting solvent; the eluates were further reduced to a final volume of 2 cm^3^ using nitrogen gas and reconstitute with 2 cm^3^ of chromatographic grade iso-octane. The purified extracts (in sealed vials) were kept in the refrigerator for analysis with Gas chromatography/ flame ionization detector (GCFID).

### Heavy metals and PAHs analysis

For metal analysis, 20 mL water sample was treated with 5 mL 69% HNO3 acid and 2 mL 30% H2O2 in a closed Teflon vessel and was digested in a Microwave Digestion System. The digested solution was then filtered through Whatman glass filter paper and the heavy metal (Arsenic, cadmium, chromium, copper, lead, nickel and zinc) contents in the water samples at different locations were measured using atomic absorption spectrophotometry (AAS; Agilent Varian AA240, USA). The metal analysis was carried out in triplicates alongside with blank to confirm accuracy of the data for quality control purposes.

Chromatographic analytical quantification was performed with 7890A Agilent gas chromatograph coupled to a flame ionization detector, Polaris Q, manufactured by Thermo Scientific. A HP-5 capillary column (30 m × 320 μm × 0.25 μm) from Agilent Technology Inc. (Santa Clara, USA) was used with an oven temperature program, starting at 60°C followed by heating at a rate of 30°C/ min to 150°C, an increase to 210°C at a rate of 12°C min−1 held for 4 min, an increase to 240°C at a rate of 15°C/ min, an increase to 320°C at a rate of 8°C/ min held for 5 min. The carrier gas was helium (99.999%) at a flow rate of 1.2 mL/ min with a make-up gas: nitrogen. Ignition gases were hydrogen and compressed air. The injector was operated at 270°C in splitless mode for 3 min. The ion trap was operated in the electronic impact mode (EI) with 70 eV energy and positive mode. The temperature of the ion source was 250°C and the interface temperature was 300°C. The analysis was performed in segment scan mode (m/z 50–300). Total run time and detector temperature were 32.25 min and 325°C respectively. The concentration of the analytes was determined by the peak area of the sample against those of the standard with which the equipment was calibrated.

### Quality control

The limit of detection (LOD) and limit of quantification (LOQ) of heavy metals was determined by three (3) times the standard deviation (SD) of sample blanks and two (2) times the LOD value [37]. The reference standards were used to determine regression (R2) of each metal using different concentration, as metal recovery ranged from 93.5% to 105% for all assessed heavy metals. The quality control measures for the PAHs analysis was in accordance to Ugochukwu et al., (38) report. The limit of detection (LOD) for the PAHs varied within the range of 0.002 and 0.124 µ g/l, whereas the limit of quantitation (LOQ) varied between 0.011 and 0.412 µ g/l. the LOD and LOQ of heavy metals and PAHs for the analyzed water samples are presented in Table 1.

### Pollution indicators

The extent of incessant heavy metals contamination in water can be evaluated by determination of contamination factor (CF), pollution load index (PLI), geo-accumulation index (I_geo_), ecological risk factor (Er), potential ecological risk index (PERI) and Nemerow index (P_N_).

### Contamination factor and pollution load index

The level of heavy metal pollution at different locations is measured using an equation proposed by Hakanson, [39].


(1) $$\mathrm{CF}=\frac{c}{c_{b}}$$

Where C is the heavy metal mean concentration in the water; C_b_ is the background value of the heavy metal. The background values of Pb, Cd, Cr, and As include 5, 0.2, 0.2 and 0.4 mg/l [39]. The proposed contamination factor has been classified into categories for monitoring pollution of metals in water for period of time: CF < 1, low contamination; 1 ≤ CF < 3, moderate contamination; 3 ≤ CF < 6, considerable contamination; and CF ≥ 6, very high contamination.


(2) $$\mathrm{PLI}=({\mathrm{CF}}_{1}\times {\mathrm{CF}}_{2}\times {\mathrm{CF}}_{3}\times \cdots \times {\mathrm{CF}}_{n}{)}^{1/n}$$

CF is the contamination factor and n is the number of heavy metals [40].

The level of pollution is classified as given: PLI < 1, no pollution; 1 < PLI < 2, moderate pollution; 2 < PLI < 3, heavy pollution; 3 < PLI, extremely high pollution.

### Geo-accumulation index (I_geo_)

The degree of geochemical accumulation of metal pollutants in water can be accessed for further pollution check by the following calculation [41].


(3) $$I_{\mathrm{geo}}={\mathrm{Log}}_{2}\;\left[\frac{c_{n}}{1.5c_{b}}\right]$$

Cn is the concentration of metal in sample; C_b_ is the geochemical background reference value of while the factor 1.5 is used due to possible variations. I_geo_ ≤ 0, uncontaminated; 0 < I_geo_ ≤ 1, uncontaminated to moderately contaminated; 1 < I_geo_ ≤ 2, moderately contaminated; 2 < I_geo_ ≤ 3; moderately to heavily contaminated; 3 < I_geo_ ≤ 4, heavily contaminated; 4 < I_geo_ ≤ 5, heavily to extremely contaminated; I_geo_ ≥ 5, extremely contaminated.

### Nemerow pollution index

The widespread effects of heavy metals and its interpretation at a particular water environment are revealed using Nemerow pollution index [42]. The equation for calculating Nemerow pollution index (P_N_) is as follows:


(4) $$P_{N}=\sqrt{\frac{{{\bar{C}}_{f}}^{2}+C_{fmax}^{2}}{2}}$$

P_N_ is the Nemerow pollution index, C_f_ the arithmetic mean of contamination factor of all heavy metals, and C_fmax_ the maximum contamination factor among the heavy metals.

### Potential ecological risk index (PERI)

The PERI is specially proposed to assess the contamination levels of surface and ground water with respect to toxicity of some selected contaminants [43][44].


(5) $$\mathrm{PERI}=\sum_{}^{}Er$$

(6) Er=Tri×CFi

Tri represents toxicity response coefficient for individual heavy metal. The toxic-response factors for Cr, As, Cd and Pb were 2, 10, 30 and 5, respectively [45][46]. Er < 40, low ecological risk; 40 ≤ Er < 80, mild ecological risk; 80 ≤ Er < 160, considerable ecological risk; 160 ≤ Er < 320, high ecological risk; Er > 320, very high ecological risk. PERI < 110, low potential ecological risk; 110 ≤ PERI < 200, moderate potential risk; 200 ≤ PERI < 400, considerable potential risk; PERI ≥ 400, very high potential risk [39].

### Health risk assessment study of heavy metals

#### Chronic daily intake (CDI)

The chronic daily consumption of heavy metals through ingestion and dermal pathway was estimated for public health assessment as given in equation 4 and 5 [30][47][48];


(7) $$\mathrm{CDI}\;(\mathrm{for}\;\mathrm{ingestion})\;=\;\frac{C\times \mathrm{EF}\times \mathrm{ED}\times \mathrm{IR}}{\mathrm{BW}\times \mathrm{AT}\times 10^{6}}$$

(8) $$\mathrm{CDI}\;(\mathrm{for}\;\mathrm{dermal}\;\mathrm{contact})\;=\;\frac{C\times \mathrm{SA}\times \mathrm{KC}\times \mathrm{EF}\times \mathrm{ET}\times \mathrm{ED}\times \mathrm{ABS}}{\mathrm{BW}\times \mathrm{AT}\times 10^{6}}$$

Where C is heavy metal concentration (mg/l) in water; EF is exposure frequency: 365 d/y [49]; IR is ingestion rate: 2 l/d for adult and 1l/d for child; ED is exposure duration: 70 years for adult and 6 years for the child [49]; BW is body weight: 60 kg for adult, 15 kg for children [49]; AT is average time: 25,550 days for adult and 2190 days for children (12, 35 in SW) ; SA is skin surface area for water contact exposure: 5700 cm^2^/d (adult), 2800 cm^2^/d (child) [49]; KC is dermal permeability factor: 0.001 for As and Cd, 0.002 for Cr and 0.004 for Pb cm/h [48]; ET is exposure time: 0.8 h/d for adult and 0.6 h/d for children; 106 was used to convert from kg to mg; ABS is fraction of dermal absorption: 0.03 (for As) and 0.001 (for others) [49].

#### Hazard quotient

The level of health risk assessment for non-carcinogenic exposure of toxic heavy metals through utilization of surface and borehole water is determined as shown in the equation reported by Oluwole et al. [50].


(9) $$\mathrm{HQ}=\frac{CDI}{RfD}$$

Where CDI is the chronic daily intake (mg kg^-1^ d^-1^); RfD is the chronic reference dose values of heavy metals (mg/kg per day): 0.0005 (RfD_ing_) and 0.000025 (RfD_derm_) for Cd; 0.0003 (both RfD_ing_ and RfD_derm_) for As; 0.003 (RfD_ing_) and 0.000075 (RfD_derm_) for Cr; 0.0014 (RfD_ing_) and 0.00042(RfD_derm_) for Pb [51,52].

#### Hazard index (HI)

Hazard index is utilized to evaluate the collective non-carcinogenic health risk exposure of dictated heavy metals emanating from different pathways. For the purpose of this study, HI is expressed as the summation of individual hazard quotient of the measured heavy metals for both ingestion and dermal absorption route [53, 54] as given in equation


(10) $$\mathrm{HI}=\sum_{i=1}^{n}HQ_{ing}+HQ_{der}$$

HI < 1 implies insignificant non-carcinogenic risk to the exposed public health but when the permissible value of HI > 1 there is tendency of non-cancer effect which might require further inspection of the surface and ground water of the study area.

#### Cancer risk

The risk of cancer exposure to health as a result of ingestion and dermal contact of heavy metals in water is estimated as follows:


(11) CR=CDI×CSF

The cancer slope factor (CSF) of Cd, As, Pb, Cr is 6.1, 1.5, 0.5 and 0.009 mg/kg per day [30][55].The lifetime cancer risk (LCR) will be calculated from the summation of cancer risk through ingestion and dermal pathway computed as follows


(12) $$\mathrm{LCR}=\sum_{}^{}CR_{ing}+CR_{der}$$

The acceptable limit for carcinogenic risk effect in water for a lifetime is within 10-6 to 10-4 in accordance with [56,57]. When CR value is above 10^-4^ then there will be high risk of cancerous disease in humans.

### Health risk assessment of PAHs

Exposure of PAHs to human health is assessed and achieved by calculating the possibility of carcinogenic and/or non-carcinogenic severe health effects to individuals over a particular period of time [6][58].

Chronic daily intake through oral and dermal exposure was estimated for non-carcinogenic risk of PAHs in contaminated water environment.


(13) $$\mathrm{CDI}-\mathrm{ingestion}\;(\mathrm{mg}/\mathrm{kg}/\mathrm{day})=\frac{\mathrm{CS}\times IR_{W}\times \mathrm{EF}\times \mathrm{ED}\times \mathrm{TF}}{\mathrm{BW}\times \mathrm{AT}}$$

(14) $$\mathrm{CDI}-\mathrm{dermal}\;(\mathrm{mg}/\mathrm{kg}/\mathrm{day})=\frac{\mathrm{CS}\times \mathrm{SA}\times \mathrm{AF}\times \mathrm{ABSsk}\times \mathrm{ETW}\times \mathrm{EF}\times \mathrm{ED}\times \mathrm{TF}}{\mathrm{BW}\times \mathrm{AT}}$$

where CS is PAHS concentration in water (mg/L), IRw is daily water ingestion rate (L/day) (2.5 L/day for adults and 0.78 L/day for children), EF is exposure frequency (350-day year-1), ED is exposure duration (26 years for adults and 6 years for children), TF is target risk (1 × 10^-6^ mg/mg), BW is body weight (80 kg for adults and 15 kg for children), AT is average time (non-carcinogens = ED ×365 days), (carcinogen = 70 × 365), SA is skin surface area (19652 cm^2^ for adults and 6365 cm^2^ for children), AF is water adherence: (0.2 mgcm^-2^ for adults and children), ABSsk is fraction of chemical absorbed through the skin (unit-less) (0.001 for adults and children), ETw is exposure time during work event (1 h/event for adults and children).

The hazard quotient (HQ) and hazard index (HI) were thereafter calculated for the individual organic pollutants.


(15) $$\mathrm{HI}={\mathrm{HQ}}_{ing}+{\mathrm{HQ}}_{der}=\left(\left[\frac{CDI_{ing}}{RfD}]+[\frac{CDI_{der}}{RfD}\right]\right)$$

HI is hazard index that is the sum of all hazard quotients (HQ) of multiple exposure pathway, HQ is probable condition that can lead to adverse health effect, CDI (E) is the chronic daily intake for any exposure matrix, RfD is reference dose (mg/kg/day) [6][59, 60].

### Carcinogenic risk assessment


(16) $$\mathrm{Cancer}\;{\mathrm{risk}}_{total}={\mathrm{Risk}}_{ing}+{\mathrm{Risk}}_{der}=\left(\left[{\mathrm{CDI}}_{\left(\mathrm{Ing}\right)}\times \mathrm{OSF}\right]+\left[{\mathrm{CDI}}_{\left(\mathrm{der}\right)}\times \mathrm{CSF}\right]\right)$$

Where cancer risk is the possibility of an adult or child developing cancer over a lifetime, CDI (E) is the chronic daily intake for any exposure matrix (ingestion, dermal and inhalation), CSF is cancer slope factor of PAHs (mg/kg/day)^-1^, and OSF is oral slope factor (mg/kg/day)^-1^ [61, 62].

The toxic equivalent quotient (TEQ) or carcinogenic potential of high molecular weight PAHs which can also be referred to as the capacity of each congener to cause modification in human’s DNA (deoxyribonucleic acid), was estimated by multiplying its individual toxic equivalent factor (TEF) with the mean concentration of each PAH in the water samples as shown in Equation [6][63].


(17) $$\mathrm{TEQ}=\sum_{}^{}Cn.\;TEFn$$

Where Cn is concentration of the individual PAH congener (n) in the mixture, and TEFn is the toxic equivalence factor for individual PAH congener (n) [64].

### Statistical analysis

The data attained from water samples were subjected to different statistical analysis using Microsoft Excel 2019 software to determine graphical charts (100% stark column with adjoining lines, pareto chart, cluster column), multivariate analysis (Pearson correlation) to derive valuable information as regards the chemical composition across sample locations.

## Results and Discussion

### PAHs composition of water samples in Ekulu River

The triplicate mean of PAHs concentration for surface water samples are presented in Table 2, as between nine (9) and eleven (11) PAHs components were detected across station A, B and C in Ekulu River, Enugu, Nigeria. The minimum and maximum value of PAHs for station A (fluorene, 0.004 mg/l; anthracene, 0.73 mg/l), station B (acenaphthene, 0.0004 mg/l; phenanthrene, 0.596 mg/l) and station C (pyrene, 0.0006 mg/l; benzo(ghi) peylene, 0.554 mg/l). the PAHs components (naphthalene, benzo(a) anthracene, chrysene, benzo(b)fluoranthene and indeno (1,2,3-cd) pyrene) were below detection levels across all stations as the cumulative PAHs components were 3.17mg/l, 1.51mg/l and 1.83 mg/l across all stations.

**Figure 2.**
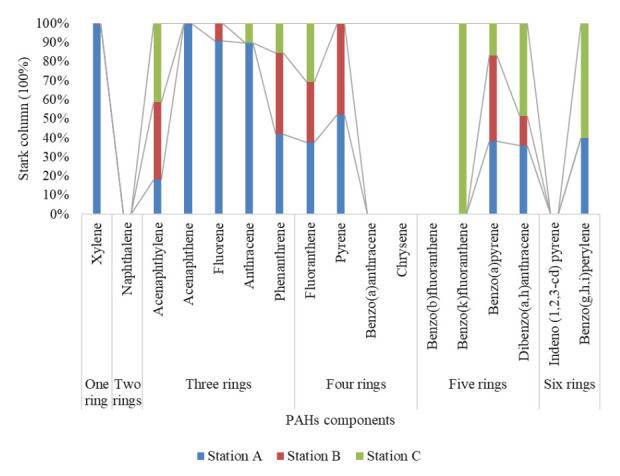
Percentage stark column of PAHs in surface water samples

Figure 2 gives the percentage stark column with trend line of PAHs components across Station A, B and C, as one can see that station A was predominant across all samples between 18.23–99.84%, which is acenaphthylene, dibenzo(a,h)anthracene; fluoranthene; benzo(a)pyrene, benzo(g,h,i)perylene; phenanthrene, pyrene, anthracene, fluorene, acenaphthene and xylene. Station B ranged between 0.16 – 47.67 %, which is xylene, acenaphthene, fluorene, dibenzo(a,h)anthracene, fluoranthene, acenaphthylene, phenanthrene, benzo(a) pyrene and pyrene, and station C ranged between 0.17 – 100%, which is pyrene, anthracene, phenanthrene, benzo(a)pyrene, fluoranthene, acenaphthylene, dibenzo(a,h)anthracene, benzo(g,h,i)perylene and benzo(k)fluoranthene.

**Figure 3.**
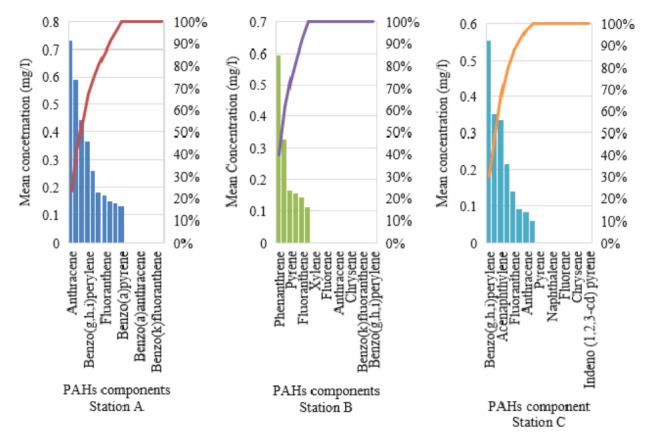
Pareto chart of PAHs components across sample locations

Pareto chart was conducted to assess the cumulative of PAHs of station A, B and C as displayed in Figure 3. In decreasing order, the cumulative PAHs of station A is anthracene, phenanthrene, xylene, benzo(g,h,i)perylene, dibenzo(a,h)anthracene, pyrene, fluoranthene, acenaphthylene, acenaphthene and benzo(a)pyrene with steady increase from 25% Pareto line towards 100%, which is invariably due to the PAHs component detected. The Pareto chart of station B and C showed an increase from 40% and 30% Pareto lines towards 100% due to PAHs assessed across variable sample locations.

The ring size were categorized with an intent to aggregate the detected PAHs composition across sampled locations (station A, B and C), so therefore, the cumulative sum of PAHs rings was arranged accordingly as shown in Table 1 and graphically presented in Figure 2, we can see that two rings were not detected (0.00 mg/l) across all samples, where as one (1), three (3), four (4), five (5) and six (6) rings were available, which gives a preview to assessing the molecular weight of PAHs components that is LMW (low molecular weight) and HMW (high molecular weight), where LMW is due to one to three rings accounting for 41.2%, while HMW (four to six rings) at 58.8% of total PAHs in water samples. The LMW levels were in higher than Ambade et al. [3], which had 48.7 in surface water. Okafor et al. [37], Zhang et al. [65] stated that LMW PAHs is due to petrogenic and combustion (low temperature pyrolysis), while HMW PAHs is mainly as a result of pyrolytic, as such the PAHs composition assessed (Table 2) implies that LMW PAHs was dominant than HMW suggesting that incomplete fossil combustion at low pyrolytic temperature in surface water from automobile and indiscriminate waste and bush burning around Ekulu river [17][66]. The LMW results were in tandem to Adeniji et al. [66] in Eastern Cape, South Africa, Hussein et al. [67] in Rea Sea, Egypt, Jamhari et al. [68] in Malaysia. In addition, LMW PAHs are least carcinogenic and harmful in relation to HMW PAHs, as seen Table 2, which implies that LMW PAHs have low solubility, high volatility and more soluble PAHs fractions and vice versa for HMW PAHs as there is possibility of aesthetics issues in terms of physiochemical parameters such as taste, odor, temperature, dissolved oxygen, BODs and CODs in surface water [26][69-71].

The total (∑) PAHs of station A, B and C were higher than World Health Organization standard (WHO) of 0.001mg/l [72] and Agency for Toxic Substances and Disease Registry (ATSDR) recommended value of 0.002mg/l [73], and lower than acceptable limit of 10 mg/l by Federal Ministry of Environment (FmE), Nigeria [74], which implies that if there is continuous increase or exposure to PAHs to different age and gender, it has the potential to cause immerse health issues to the populace who directly or indirectly utilize the surface water resource such as flora and faunas, as studies has shown that transfer potential from one ecological source is possible due to biotransformation or exposure [72][75].

A closer assessment of PAHs components showed that xylene was assessed, which is not part of PAHs but is categorized as BTEX (benzene, toluene, ethyl benzene and xylene) according to WHO [72] in terms of aromatic hydrocarbons as they are known to cause immerse negative health issues, detectable taste and odor issues, as WHO threshold level of 0.3mg/l implies that station A will have distinctive issues, but the health-based guideline value of 0.5mg/ is acceptable. Xylenes are group of BTEX used to blend petroleum product and chemical additives, which are released from low temperature pyrolysis (cigarette) and petro genic source (point emission or releases) [76, 77].

PAHs are already known as a ubiquitous micro pollutant that has potential to transform biochemically or chemically to human exposure source in relation to the ecosystem, as the benzo(a)pyrene (BaP) has been known as a reference toolkit for assessing PAHs in diverse form with lethal dose (LC50) of 0.001mg/l or 0.002 mg/l of either WHO or ASTDR reference has the potential to cause toxic response from immerse exposure to humans from a host of source points [8][64]. So therefore, incomplete combustion of agriculture and waste in relation to automobile and power generating plants within the study location has the potential to increase PAHs observed in the water body.

Table 3 shows a comprehensive correlation potential of PAHs across the three surface water sites (Station A – C) in Ekulu river, as it gives a regression pattern from a weak correlation (< 0.30) to medium correlation (0.31 – 0.69) to strong correlation (> 0.70) accordingly. We can therefore see that Xyl correlated strongly with Ace, Flu, Anth and Fluo at 0.99, Phen correlated strongly between positive and negative variables of -0.77 – 0.99 in Pyr, BkF, BaP, DBA and BghiP. Acy correlated negatively with Ace, Flu, Anth and Fluo (-0.99). A second review also shows that the PAHs ring clusters of 1 and 3 has positive correlation with with Xyl, Ace, Flu, Anth, Fluo, as PAHs of 4 rings has medium to strong correlations. For PAHs 5 and 6 rings, there was similar strong correlation in Phen, Pyr, Bkf, BaP, DBA and BghiP, which points to the facts that there are strong interactions across the various ring structure from biochemical transformation and reactions (volatilization, biodegradation, photolytic oxidation and sorption) with different environmental conditions [38]. Ring 3 correlated strongly with ring 1 and 4 at 0.96 and 0.85, while ring 5 had negative and positive correlation with ring 4 and 6 at -0.73 and 0.99 respectively. As regards HMW and CPAHs, there was strong correlation at 0.97 indicating vehicular emissions (petrogenic PAHs), as total (∑) PAHs correlated with LMW and HMW at 0.93 and 0.55, which complies with earlier statement that LMW will have petrogenic and low pyrolysis (combustion) of carbonaceous compounds as seen with other PAHs components assessed [6][68].

So therefore, strong correlation implies that PAHs components in water resources has common pollution source from a host of anthropogenic activities emanating from dumping of waste to the river, agricultural and runoff from industries, as medium to weak correlation implies that there is slight or no pollution influence on the aquatic ecosystem, which is in tandem to seasonal variations as stated by Ojaniyi et al. [78]. For positive and negative correlation, which was dominant across all accessed principal component analysis, shows that positive correlation is predominant in similar reaction mode or biochemical transformation in the water bodies and vice versa for negative correlation, which could be side reactions from other environmental pollutants such as heavy metals, organometallics, and BTEX components in relation to conditions such as thermal decomposition and microbial interactions in aquatic resources [76].

### Molecular Diagnostic of PAHs

The molecular diagnostics of PAHs were assessed in Table 4 to assess the ratio of individual PAHs components in relation to source or origin identification of PAHs. The ratios assessed were used to distinguish petrogenic (unburnt crude oil, gas and coal), pyrogenic combustion of carbonaceous components (natural and anthropogenic) and phytogenic (plants, animal and microbial decomposition). As seen in Table 4, the ratio of Phen/Ant and Anth/(Anth+Phen) points towards pyrogenic, Fluo/(Pry+Fluo), Anth/178 reveals petrogenic and Fluo/Pyr, LMW/HMW and CarC. PAHs/Total PAHs in the water station A, B and C points to a mixed source (petrogenic, pyrogenic and phytogenic). According to Zhang et al. [65], Anth/(Anth+Phen) and Anth/178 indicates pyrolytic combustion or carbonization of natural and anthropogenic sources if greater than 0.1, as we can see that there is a mixture of petrogenic and pyrogenic from station A, B and C correspondingly. Adeniji et al. [6] molecular diagnostics confirms Flt/Pyr, LMW/HMW assessed in this study due to natural and anthropogenic sources.

### Ecological Risk Indices of PAHs

**Figure 4.**
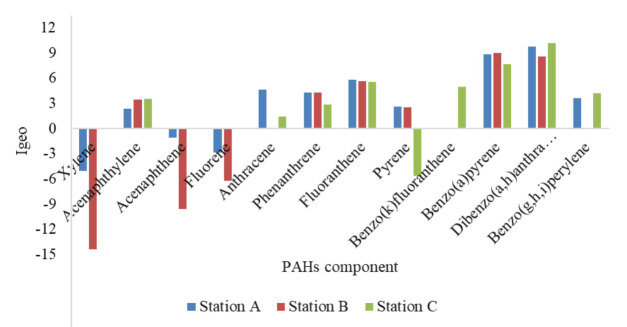
Geoaccumulation index of PAHs components

The ecological risk indices were conducted using US EPA minimum contamination levels (MCL) to assess the influence of PAHs in water bodies. Table 5 shows the ecological indices assessed, which are contamination factor (CF), pollution load index (PLI) and Nemerow pollution index (NPI) of water samples in Ekulu River. The contamination factor (CF) shows that the minimum and maximum value of stations A, B and C are 4.43E-02 – 1.30E+03; 7.00E-05 – 7.74E+02; 3.00E-02 – 1.76E+02 with number of variables of eleven (11), nine (9) and nine (9) indicating high level of contamination across all stations assessed. The pollution load index (PLI) were assessed across station A, B and C using the standard that is greater than one (1) representing PAHs pollution, as such all cumulative PAHs components were extremely polluted [37][78]. Nemerow pollution index (NPI) conducted using reference guide of unpolluted (0.00 – 1.00), slightly polluted (>1.00 – 2.5), moderately polluted (> 2.5 – 7.0), highly polluted (>7.0), so therefore, all stations were highly polluted and lead to high bioaccumulation of PAHs components in aquatic environment in Ekulu river, Enugu, Nigeria [42]. Aquatic environment acts as a multisite to dilution, transformation, biochemical and physical interacts of diverse pollutants, on a case to case basis. The clustered column (Figure 4) assessed produced a combination of positive and negative I_geo_ values using reference guide as depicted by Zhang et al., [79] showed that station A, B and C of fluoranthene, benzo(a)pyrene and dibenzo(a,h) anthracene were extremely contaminated for positive I_geo_, as other samples were between low and moderate contamination while station B of xylene, acenaphthene and fluorene had negative none contamination. According to Latosinska et al., [80] contaminants above certain concentration from a host of anthropogenic sources has the capacity to geologically accumulate based on point source releases causing biotransformation and exposure to various organisms (including humans) thereby affecting their health and wellbeing.

### Health risk assessment of PAHs

**Figure 5.**
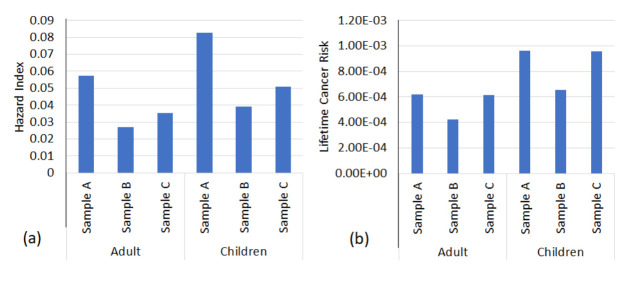
(a) Cumulative hazard index of PAHs in adult and children; (b) and lifetime cancer risk of PAHs in adult and children

The degree of human exposure cannot be overstated, as it is a known fact that diverse activities has the ability to either affect our health or wellbeing or not, as the case may suffice. Hazard index and cancer risk as known US Environmental protection terminologies that has been effectively developed since inception, as it gives a relative non-cancer and cancer impact from long- or short-term exposure is dependent on the actual PAHs concentration from a host of exposed medium such as oral, dermal and inhalation that is dependent on the exposure source (atmosphere, hydrosphere and lithosphere). So therefore, we can see that for hydrosphere (aquatic or water body) exposure, oral and dermal exposure was conducted using USEPA calculation matrices (adult and children) and cumulated to get hazard quotient or cancer risk of the aforementioned, thereafter, they were summed to get the hazard index and cumulative cancer risk as shown in Table 6 in tandem to Figure 5, which is the summation of hazard index and cancer risk.

Hazard quotient or hazard index less than one (1) means that there is no significant health issues or noncarcinogenic risk associated with either adults or children in a given population collection [49]. The hazard index obtained from the studied area (Table 6a), showed that dermal condition were dominant than oral exposure, which was all less than one (1), implying that both adult and children were within safe levels, as ∑PAHs (Figure 5a) of station A, B and C are 0.057 and 0.082; 0.027 and 0.039; 0.035 and 0.05, which were below USEPA recommendation limit [81, 82].

Cancer risk was conducted as shown in Table 6b, where the CSF (cancer slope factor) was evaluated with CDI (chronic daily intake) to get the values for the population matrices (adults and children). The lifetime cancer risk (Figure 5b), which is the cumulative cancer risk for one in a million population (1⁄1,000,000) seen as US-EPA acceptable limit but values less than one in a thousand population (1⁄10,000) is known to cause serious cancer risk in humans [6][76]. As we can see, benzo(a)pyrene and dibenzo(a,h)anthracene was in the range of 8.61×10^-5^ to 1.66×10^-4^ and 8.06×10^-4^ to 1.34×10^-4^ indicating significant cancer risk for adult and children.

Having assessed the study locations, one can therefore state that children are prone to having non-carcinogenic (adverse health effect) and carcinogenic (cancer based issues) over a period of time usually 70 years as stipulated by USEPA from continuous exposure to anthropogenic activities, as WHO and US-EPA has stated that although PAHs is excreted from the human body via urination or sweating, it still has the ability to impact on the health and wellbeing of children more than adults, but we need to know that if a child is exposed for a long period from birth, it has the potential to weaken the body defenses against potential PAHs exposure thereby causing immerse health based illness.

### Toxic Equivalent Quotient of PAHs in surface water

**Figure 6.**
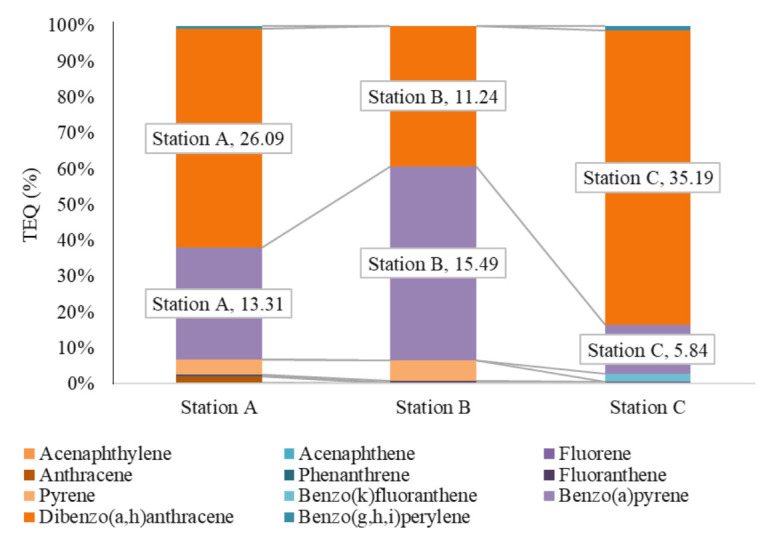
Toxic equivalent quotient of PAHs components across sample stations

The estimated carcinogenic potentials (toxic equivalent quotient) of 11 PAHs components were evaluated using CCME, [66] reference standard multiplied by PAHs concentration to get the toxic equivalent quotient (TEQ) as shown in Table 7 and Figure 6. The total TEQ (%) of station A, B and C are 42.70, 28.63 and 42.78, where benzo(a)pyrene and dibenzo(a,h)anthracene, carcinogenic PAHs contributed to the highest TEQ between 11.24% to 35.19% as seen in Figure 6. The cumulative TEQ of PAHs in Ekulu river means that benzo(a)pyrene and dibenzo(a,h)anthracene will contribute and trigger a number of health issues such as neurobehavioral impairment, respiratory issues, birth deformities, reproductive impotence, dermatological and many other in the human body [76][83, 84], as other PAHs did not record any substantial contribution, hence the cumulative potential risk is considerably minimal across samples stations in Ekulu river.

### Heavy metals composition of surface water

**Figure 7.**
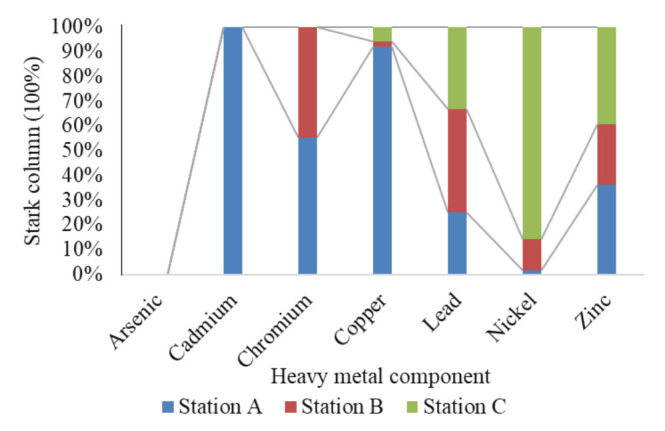
Percentage stark column of heavy metals in surface water samples

**Figure 8.**
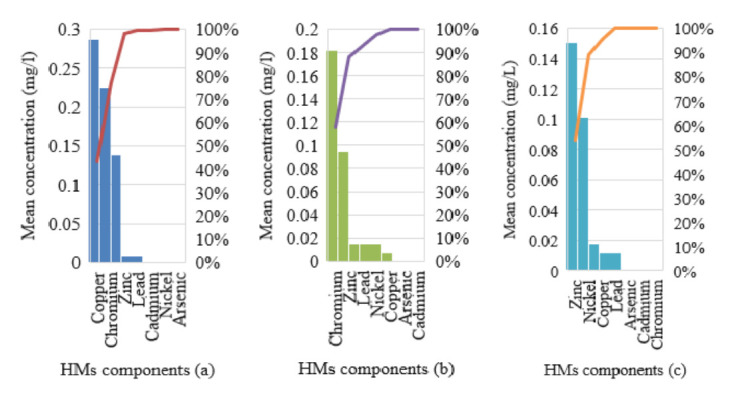
Pareto chart of heavy metals across surface water samples

The total mean concentration of heavy metals is presented in Table 8, as we can see that arsenic was absent across the three sampled stations A, B and C, which could be low detection limit of instruments, which is in agreement with previous studies conducted by Ugochukwu et al. [31] in urban groundwater and water supply system in Enugu state, Nigeria. Cadmium was present in station A only, which was within USEPA and WHO limit of 0.005mg/l and 0.003mg/l. Chromium was present in station A and B, which were above USEPA and WHO limit of 0.1mg/l and 0.05mg/l, as other metals (copper, lead, nickel, zinc) were present in all samples. The mean copper, nickel and zinc were within USEPA and WHO standards, while mean lead concentration of station A were above regulated levels, as B and C were with limits. Figure 7 gives the stark column aggregates of HMs in surface water from Ekulu that showed that station A was predominant in decreasing order of cadmium, copper, chromium, zinc, lead and nickel, as station C was next with decreasing order of nickel, zinc, lead and copper and station B having HMs of chromium < lead < zinc < nickel < copper respectively. Stark column gives a percentile value of aggregated groups for easy identification and description across a study range of 0 – 1 (0% – 100%). Figure 8 shows Pareto chart of HMs in Ekulu water samples that were assessed, we can see that station A (a), B (b) and C (c) showed a steady increase from 44%, 58% and 53% to 100%, which were due to the relative closeness and interactions of HMs variables (mean concentrations) assessed in tandem to the different sampling stations.

A review of assessed heavy metals (HMs) showed that arsenic was absent, as such there will be no significant health issues that can impact Ekulu inhabitants provided that contamination does not take place in the future from a number of exposures, but arsenic forms various organic and inorganic compounds (metal sulfides or arsenates or arsenides) with different oxidation states and toxicity in terms of its physiochemical interactions and properties across different ecology [72], which is known to cause number of ailments such as dermal lesions and cancer, bladder and lungs cancer, cardiovascular and neurological health issues from chronic arsenicism intake in children predominantly and adult in the future [79][85]. Cadmium is adsorbed into the kidney, which have biochemical half-life of 10 – 35 years, as the International agency for research on cancer (IARC) has associated cadmium as a carcinogen with presence of tubular dysfunction across an exposed population [72]. Chromium exist predominantly in valences of +3 and +6 [78], as Cr+3 is an essential nutrient, while Cr+6 is known as a carcinogen, which is known to cause tumor but WHO [72] suggest that Cr+6 can be reduced to Cr+3 in the gastrointestinal tract and stomach compartment at low dose and excreted via urination [78, 85]. Copper is known as an essential nutrient that is utilized for metabolic, enzymatic and ionic process in the human body, as concentration above USEPA and WHO limit of 1.30mg/l and 2mg/l is known to cause gastric irritation, diarrhea and nausea from short term oral exposure, which is dependent on concentration and age in a population matrix, while long term exposure leads to liver and kidney damage, as it is known to cause a taste (bitter) and color is affected at extreme concentration above 2mg/l [86-88]. Lead is also a toxic metal that cause neurological effect, nephrological effect, reproductive issues, and other health issues such as vitamin D inhibition, restlessness and sleeplessness in children, acute psychosis, anemia (low red blood cell production), impaired vision and hearing from both long- and short-term exposures [30]. Nickel exposure is known to cause eczematic reaction (allergic dermatitis) in both children and adults in relation to liver and kidney damage [89]. Zinc is an essential metal that act as a biomechanical component for life [85], as Ukah et al. [87] stated that extreme concentration has the potential to cause undesirable taste issues, fatigue, nausea, brain dysfunction and malaise (fever) over a long-term exposure.

### Source identification of HMs across all sample locations

The correlation between different HMs parameters were assessed on the cumulative mean concentration of station A, B and C as presented in Table 9. The correlation was significant at p < 0.05, which were conducted using Pearson correlation using the aggregation of weak correlation (< 0.30), medium correlation (0.31 – 0.69) and strong correlation (> 0.70) accordingly. Cadmium correlated positively with chromium (0.65) and copper (0.99), which can be associated with industrial wastewater and effluents and waste leachate, as it correlated negatively with lead (-0.87) and nickel (-0.60) implying difference in their origin and interaction [90, 91]. Lead correlated negatively with copper (-0.88), which suggests that they were not from similar pollution source, as nickel had similar correlation with chromium (-0.99) and copper (-0.57). So therefore, we can see that positive correlation indicates that they are from similar source, biotransformation and physiochemical interactions, which is vice versa for negative correlations.

### Ecological risk indices of HMs in Ekulu River

**Figure 9.**
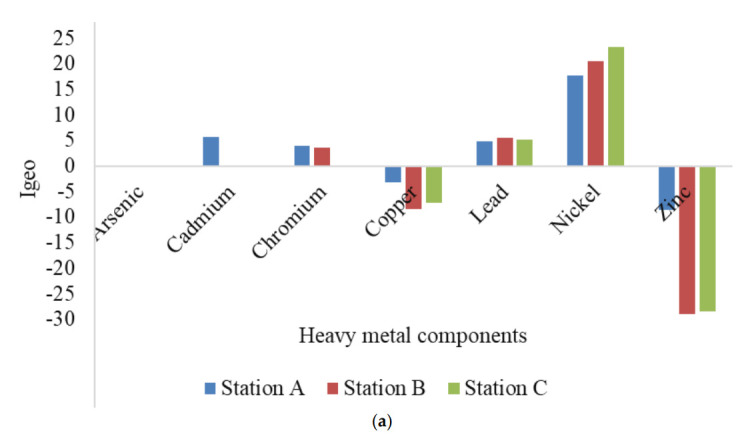
Geoaccumulation index of Heavy metals in surface water samples

Table 10 shows the ecological indices of Ekulu river that was assessed for contamination factor (CF), pollution load index (PLI), Nemerow pollution index (NPI), potential ecological risk indices (PERI) and geoaccumulation index (I_geo_). The contamination factor (CF) of heavy metals were calculated to assess the influence of anthropogenic activities on water samples, as it was found to follow decreasing levels (Table 9): station A (Ni > Cr > As > Cu > Cd > Pb > Zn), station B (Ni > Cr > Pb > As > Cd > Cu > Zn) and station C (Ni > As > Cd > Cr > Pb > Cu > Zn). The PLI value close to one (< 1) indicate no pollution and vice versa for values greater than one (> 1), as such, all station was less than one indicating no pollution. The NPI values using reference guide showed that station A was moderately polluted while B and C were highly polluted across the cumulative HMs components in Ekulu River. The PERI was conducted as shown in Table 9, where station A, B and C was 6.29, 38.61 and 252.66, which implies that station A and B were low potential ecological risk (< 110), while station C was moderate potential ecological risk (200 ≤ PERI < 400), which was dependent on the cumulative HMs assessed accordingly, thus indicating that metal concentration above reasonable standards have the potential to produce high bioaccumulation in the environment thereby posing health risk to humans [20]. Figure 9 gives the geoaccumulation index (I_geo_) that compares the HM elements with its standard background value in accessing how the study area is polluted than its background mineral concentration in logarithmic base value of 2.0 [85]. The I_geo_ were categorized using seven different classes ranging from 0 – 6, as we can see that arsenic was 0 indicating uncontaminated. Cadmium was extremely contaminated (≥ 5) for station A only, while chromium was heavily contaminated (≥ 3) for station A and B. For copper and zinc, it was negative across all samples, which signifies no contamination, while lead and nickel were between heavily to extremely contamination across all samples.

Hence, the results of CF, PLI, NPI, PERI and I_geo_ revealed that HM pollution in Ekulu river is between moderate to heavy pollution, which showed significant study with Pal and Maiti, [85] and Rana et al., [92] in the tropics of India. The present study suggests that bioaccumulation and biotransformation of heavy metals (HMs) has the potential to either increase or decrease from a host of anthropogenic activities from rural/urban settlement to vehicular to artisanal to industrial effluents releases.

### Health risk assessment of HMs

**Figure 10.**
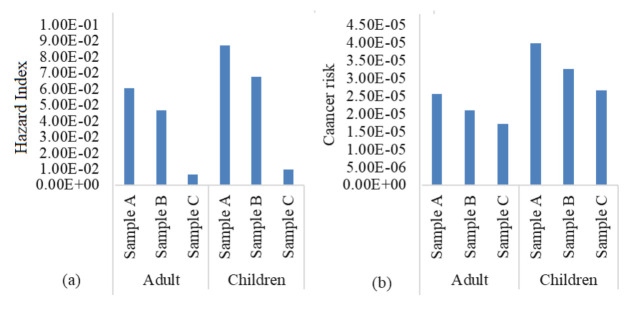
(a) Cumulative hazard index of HMs in adult and children (b) Lifetime cancer risk of HMs in adult and children

The heavy metals components were assessed using USEPA risk models to ascertain hazard index (HI) and lifetime cancer risk (LCR), as shown in Table 11 and Figure 10. Two exposure media, which are oral and dermal route were utilized, as the chronic daily intake (CDI) of dermal route was highest for both adult and children population than oral route from a host of activities that was subsequently evaluated using reference dose (RfD and cancer slope factor (CSF) for cumulative HI and LCR determination accordingly. The calculated value of cumulative HI for adults and children as follows: station A (0.061; 0.087), station B (0.047; 0.067) and station C (0.0067; 0.0096), which indicates an inconsequential non-carcinogenic health effect for both adults and children across the total HMs in surface water (Figure 10a).

The cumulative LCR of adults and children are station A (2.56 × 10^-5^; 3.98 × 10^-5^), station B (2.09 × 10^-5^; 3.26 × 10^-5^) and station C (1.72 × 10^-5^; 2.67 × 10^-5^), which were within USEPA standard of 1 × 10^-6^ – 1 × 10^-4^ in contrary to Shafiuddin Ahmed et al., [30] reports. The evaluated HI and LCR shows that children are susceptible to non-carcinogenic and carcinogenic health issues than adults because of their immunity levels are still in developmental levels that can be disrupted and are sensitive targets to these aforementioned toxic elements. In addition, these metals can readily adsorbed and distributed to body tissues and organs to cellular medium causing immerse heath issues over a period of time; thus constant monitoring of these toxic metals is required to safeguard the wellbeing of children development to adulthood.

## Conclusions

Numerous cancerous effects have been recorded through incessant exposure to organic and inorganic contaminated surface water. The present study revealed the mean concentration of polycyclic aromatic hydrocarbons (PAHs) and selected heavy metals (HMs) in Ekulu River, Enugu state. PAHs showed presence of high molecular weight PAHs (58.8%) and low molecular weight PAHs (41.2%), which was predominantly from anthropogenic activities around the study area due to pyrogenic next to petrogenic and phytogenic. Pearson correlation showed that the number of PAHs ring aggregation gives a close relationship between PAHs components, it's interaction and biotransformation from 0.0 – 1.0 (weak to strong correlation). The molecular diagnostics was in agreement that all PAHs components assessed were from pyrolytic sources than petrogenic sources. The ecological risk indices of PAHs were between low to high pollution across all sample stations, which is in tandem with anthropogenic releases. The toxic equivalent quotient of PAHs showed that benzo(a)pyrene and dibenzo(a,h)anthracene contributed to the cumulative carcinogenic levels in Ekulu river, although health risk levels were minimal across other PAHs components. Heavy metals assessment showed that arsenic was absent across all sample sources, as other parameters (cadmium, chromium, copper, nickel, and zinc) were within USEPA and WHO standard except for lead metal respectively. The source identification showed positive correlation implying a similar pollution source and vice versa for negative correlation, as ecological risk indices suggest moderate to heavy pollution across all sample source. The health risk exposure showed that children are likely to have carcinogenic and non-carcinogenic health illnesses from a host of exposures at extended period. There is need to advocate for further environmental evaluation across all sampled location. The research findings therefore give a focal point for policy makers, environmentalist, medical practitioners and advocates that the health and wellbeing of the environment is paramount for the survival of a population, as further research is needed to assess any impending health issues from a medical standpoint in Ekulu and its environs

## Figures and Tables

**Table 1.** Limit of detection and limit of quantification of heavy metals and PAHs in cumulative water sample.

Heavy metals	R^2^	LOD (mg/l)	LOQ (mg/l)	Recovery (%)
As	0.9973	0.19	0.39	94.50
Cd	0.9978	0.15	0.30	93.50
Cr	0.9961	1.25	2.50	96.30
Cu	0.9949	0.31	0.62	101.25
Pb	0.9994	0.27	0.54	98.45
Ni	0.9982	0.22	0.44	105.00
Zn	0.9993	0.004	0.006	99.05
**PAHs components**				
Xylene	0.9975	0.025	0.061	91.45
Naphthalene	0.9890	0.015	0.052	96.52
Acenaphthylene	0.9990	0.008	0.028	91.46
Acenaphthene	0.9985	0.020	0.068	86.01
Fluorene	0.9721	0.021	0.071	99.91
Anthracene	0.9970	0.004	0.019	97.12
Phenanthrene	0.9965	0.003	0.012	87.34
Fluoranthene	0.9850	0.006	0.020	99.15
Pyrene	0.9750	0.014	0.043	97.46
*Benzo(a)anthracene	0.9914	0.004	0.015	98.50
*Chrysene	0.9645	0.003	0.011	88.91
*Benzo(b)fluoranthene	0.9930	0.002	0.059	95.97
*Benzo(k)fluoranthene	0.9900	0.024	0.081	87.80
*Benzo(a)pyrene	0.9955	0.013	0.042	91.92
*Dibenzo(a,h)anthracene	0.9875	0.124	0.412	83.94
*Indeno (1,2,3-cd) pyrene	0.9626	0.061	0.203	94.59
*Benzo(g,h,i)perylene	0.9851	0.113	0.377	98.23

**Table 2.** Polycyclic Aromatic Hydrocarbons (PAHs) mean concentration of water samples

PAHs components	Station A	Station B	Station C
Xylene	4.43E-01	7.00E-04	BDL
Naphthalene	BDL	BDL	BDL
Acenaphthylene	1.48E-01	3.30E-01	3.35E-01
Acenaphthene	1.42E-01	4.00E-04	BDL
Fluorene	4.00E-03	4.00E-04	BDL
Anthracene	7.30E-01	BDL	8.26E-02
Phenanthrene	5.90E-01	5.96E-01	2.16E-01
Fluoranthene	1.70E-01	1.44E-01	1.40E-01
Pyrene	1.82E-01	1.66E-01	6.00E-04
*Benzo(a)anthracene	BDL	BDL	BDL
*Chrysene	BDL	BDL	BDL
*Benzo(b)fluoranthene	BDL	BDL	BDL
*Benzo(k)fluoranthene	BDL	BDL	9.16E-02
*Benzo(a)pyrene	1.33E-01	1.55E-01	5.84E-02
*Dibenzo(a,h)anthracene	2.61E-01	1.12E-01	3.52E-01
*Indeno (1,2,3-cd) pyrene	BDL	BDL	BDL
*Benzo(g,h,i)perylene	3.69E-01	BDL	5.54E-01
One ring	4.43E-01	7.00E-04	0.00E+00
Two rings	0.00E+00	0.00E+00	0.00E+00
Three rings	1.61E+00	9.27E-01	6.34E-01
Four rings	3.52E-01	3.10E-01	1.41E-01
Five rings	3.94E-01	2.67E-01	5.02E-01
Six rings	3.69E-01	0.00E+00	5.54E-01
LMW PAHs	2.06E+00	9.28E-01	6.34E-01
HMW PAHs	1.12E+00	5.78E-01	1.20E+00
*Carc. PAHs	7.63E-01	2.67E-01	1.06E+00
∑ PAHs	3.17E+00	1.51E+00	1.83E+00

BDL: < 0.001; LMW: low molecular weight; HMW: high molecular weight; *Carc PAHs: carcinogenic PAHs.

**Table 3.** Pearson correlation of PAHs components

	Xyl	Acy	Ace	Flu	Anth	Phen	Fluo	Pyr	BkF	BaP	DBA	BghiP	1 ring	3 rings	4 rings	5 rings	6 rings	LMW	HMW	CPAHs	ΣPAHs
Xyl	1																				
Acy	**-0.99**	1																			
Ace	**0.99**	**-0.99**	1																		
Flu	**0.99**	**-0.99**	**0.99**	1																	
Anth	**0.99**	**-0.99**	**0.99**	**0.98**	1																
Phen	0.49	-0.51	0.49	0.57	0.40	1															
Fluo	**0.99**	**-0.99**	**0.99**	**0.99**	**0.97**	0.60	1														
Pyr	0.57	-0.59	0.57	0.64	0.48	**0.99**	0.67	1													
BkF	-0.50	0.52	-0.50	-0.58	-0.41	**-0.99**	-0.61	**-0.99**	1												
BaP	0.30	-0.32	0.30	0.39	0.20	**0.98**	0.42	**0.96**	**-0.98**	1											
DBA	0.14	-0.11	0.14	0.05	0.24	**-0.80**	0.01	**-0.74**	**0.79**	**-0.90**	1										
BghiP	0.19	-0.17	0.19	0.10	0.29	**-0.77**	0.06	**-0.70**	**0.76**	**-0.88**	**0.99**	1									
1 ring	**0.99**	**-0.99**	**0.99**	**0.99**	**0.99**	0.49	**0.99**	0.58	-0.50	0.30	0.14	0.19	1								
3 rings	**0.96**	**-0.96**	**0.96**	**0.98**	**0.92**	**0.72**	**0.99**	**0.78**	**-0.73**	0.57	-0.16	-0.11	**0.96**	1							
4 rings	0.65	-0.67	0.66	**0.72**	0.57	**0.98**	**0.74**	**0.99**	**-0.98**	**0.92**	-0.66	-0.62	0.65	**0.85**	1						
5 rings	0.05	-0.02	0.04	-0.05	0.15	**-0.85**	-0.08	**-0.80**	**0.84**	**-0.94**	**0.99**	**0.99**	0.05	-0.25	**-0.73**	1					
6 rings	0.19	-0.17	0.19	0.10	0.29	**-0.77**	0.06	**-0.70**	**0.76**	**-0.88**	**0.99**	**0.99**	0.19	-0.11	-0.62	**0.99**	1				
LMW	**0.98**	**-0.99**	**0.98**	**0.99**	**0.96**	0.65	**0.99**	**0.72**	-0.66	0.48	-0.06	-0.01	**0.98**	**0.99**	**0.79**	-0.15	-0.01	1			
HMW	0.39	-0.37	0.39	0.31	0.48	-0.61	0.27	-0.54	0.60	**-0.76**	**0.97**	**0.98**	0.39	0.11	-0.44	**0.94**	**0.98**	0.20	1		
CPAHs	0.15	-0.12	0.14	0.06	0.25	**-0.79**	0.02	**-0.73**	**0.78**	**-0.90**	**0.99**	**0.99**	0.15	-0.15	-0.65	**0.99**	**0.99**	-0.05	**0.97**	1	
∑PAHs	**0.98**	**-0.98**	**0.98**	**0.96**	**0.99**	0.32	**0.95**	0.41	-0.33	0.12	0.32	0.37	**0.98**	**0.88**	0.50	0.23	0.37	**0.93**	0.55	0.33	1

Xyl: Xylene; Acy: Acenaphthylene; Ace: Acenaphthene; Flu: Fluorene; Anth: Anthracene; Phen: Phenanthrene; Fluo: Fluoranthene; Pyr: Pyrene; BkF: Benzo(k)fluoranthene; BaP: Benzo(a)pyrene; DBA: Dibenzo(a,h)anthracene; BghiP: Benzo(g,h,i)perylene.

**Table 4.** Molecular diagnostics of PAHs

PAHs components	Petrogenic	Pyrogenic	Phytogenic	Station A	Station B	Station C
Phen/Anth	> 15	< 10	–	0.8703	No Data	2.3936
Anth / (Anth + Phen)	< 0.1	> 0.1	–	0.5347	0.304	0.2947
Fluo / (Pry + Flt)	< 0.4	> 0.4	> 0.5	0.3528	0.0035	No Data
Fluo / Pyr	< 1.0	> 1.0	–	0.545	0.0036	No Data
Anth / 178	< 0.1	≥ 0.1	–	0.001	0.0008	0.0008
LMW / HMW	< 0.2	> 0.2	–	1.9189	2.4697	0.8963
CarC. PAHs / ∑ PAHs			–	0.2156	0.2133	0.3351

Phen: phenanthrene, Anth: anthracene, Fluo: fluoranthene, Pry: pyrene, LMW: low molecular weight, HMW: high molecular weight; CarC. PAHs: Carcinogenic PAHs, Σ PAHs: cumulative/total PAHs [65,66].

**Table 5.** Ecological indices of PAHs in surface water samples

PAHs components	Station A	Station B	Station C	USEPA MCL value (mg/L)
Xylene	4.43E-02	7.00E-05	No Data	1.00E+01
Acenaphthylene	7.42E+00	1.65E+01	1.68E+01	2.00E-02
Acenaphthene	7.11E-01	2.00E-03	No Data	2.00E-01
Fluorene	2.00E-01	2.00E-02	No Data	2.00E-02
Anthracene	3.65E+01	No Data	4.13E+00	2.00E-02
Phenanthrene	2.95E+01	2.98E+01	1.08E+01	2.00E-02
Fluoranthene	8.52E+01	7.21E+01	7.00E+01	2.00E-03
Pyrene	9.09E+00	8.31E+00	3.00E-02	2.00E-02
Benzo(k)fluoranthene	0.00E+00	No Data	4.58E+01	2.00E-03
Benzo(a)pyrene	6.66E+02	7.75E+02	2.92E+02	2.00E-04
Dibenzo(a,h)anthracene	1.30E+03	5.62E+02	1.76E+03	2.00E-04
Benzo(g,h,i)perylene	1.85E+01	No Data	2.77E+01	2.00E-02
N	11	9	9	
PLI	1.18E+01	1.92E+00	2.16E+01	
NPI	2.91E+01	2.34E+01	3.36E+01	

MCL: minimum contamination level; N: number of available variables; PLI: pollution load index; NPI: nemerow pollution index.

**Table 6.** Hazard Index and lifetime cancer risk of PAHs in surface water samples

HI (a)		Adult			Children		
PAHs components	RfD	Station A	Station B	Station C	Station A	Station B	Station C
Xylene	0.2	1.57E-03	2.47E-06	No Data	2.25E-03	3.56E-06	No Data
Acenaphthylene	0.06	1.75E-03	3.89E-03	3.95E-03	2.51E-03	5.60E-03	5.68E-03
Acenaphthene	0.006	1.67E-02	4.71E-05	No Data	2.41E-02	6.78E-05	No Data
Fluorene	0.04	7.07E-05	7.07E-06	No Data	1.02E-04	1.02E-05	No Data
Anthracene	0.3	1.72E-03	No Data	1.95E-04	2.48E-03	No Data	2.80E-04
Phenanthrene	0.04	1.04E-02	1.05E-02	3.81E-03	1.50E-02	1.52E-02	5.49E-03
Fluoranthene	0.04	3.01E-03	2.55E-03	2.47E-03	4.33E-03	3.67E-03	3.56E-03
Pyrene	0.03	4.28E-03	3.92E-03	1.41E-05	6.16E-03	5.64E-03	2.03E-05
Benzo(k)fluoranthene	0.03	No Data	No Data	2.16E-03	No Data	No Data	3.11E-03
Benzo(a)pyrene	0.03	3.14E-03	3.65E-03	1.38E-03	4.51E-03	5.25E-03	1.98E-03
Dibenzo(a,h)anthracene	0.03	6.15E-03	2.65E-03	8.29E-03	8.85E-03	3.81E-03	1.19E-02
Benzo(g,h,i)perylene	0.03	8.70E-03	No Data	1.31E-02	1.25E-02	No Data	1.88E-02
∑PAHs		5.75E-02	2.72E-02	3.54E-02	8.28E-02	3.93E-02	5.08E-02
**LCR (b)**		**Adult**			**Children**		
**PAHs components**	**CSF**	**Station A**	**Station B**	**Station C**	**Station A**	**Station B**	**Station C**
Xylene	NA	No CSF	No CSF	No Data	No CSF	No CSF	No Data
Acenaphthylene	0.0073	2.19E-07	4.87E-07	4.94E-07	3.40E-07	7.56E-07	7.68E-07
Acenaphthene	0.073	2.10E-06	5.90E-09	No Data	3.26E-06	9.17E-09	No Data
Fluorene	NA	No CSF	No CSF	No Data	No CSF	No CSF	No Data
Anthracene	NA	No CSF	No Data	No CSF	No CSF	No Data	No CSF
Phenanthrene	NA	No CSF	No CSF	No CSF	No CSF	No CSF	No CSF
Fluoranthene	0.073	2.51E-06	2.13E-06	2.06E-06	3.90E-06	3.30E-06	3.21E-06
Pyrene	0.73	2.68E-05	2.45E-05	8.84E-08	4.17E-05	3.81E-05	1.37E-07
Benzo(k)fluoranthene	0.0073	No Data	No Data	1.35E-07	No Data	No Data	2.10E-07
Benzo(a)pyrene	7.3	1.96E-04	2.28E-04	8.61E-05	3.05E-04	3.55E-04	1.34E-04
Dibenzo(a,h)anthracene	7.3	3.85E-04	1.66E-04	5.19E-04	5.98E-04	2.58E-04	8.06E-04
Benzo(g,h,i)perylene	0.073	5.44E-06	No Data	8.17E-06	8.46E-06	No Data	1.27E-05
LCR		6.18E-04	4.21E-04	6.16E-04	9.61E-04	6.55E-04	9.57E-04

No Data: Analytical data unavailable; No CSF: cancer slope factor unavailable; HI: Hazard index LCR: lifetime cancer risk

**Table 7.** Toxic Equivalent quotient (TEQ) of PAHs in water samples from Ekulu river

PAHs components	Station A	Station B	Station C	TEF standard
Acenaphthylene	1.48E-04	3.30E-04	3.35E-04	1.00E-03
Acenaphthene	1.42E-03	4.00E-06	No Data	1.00E-02
Fluorene	4.00E-06	4.00E-07	No Data	1.00E-03
Anthracene	7.30E-03	No Data	8.26E-04	1.00E-02
Phenanthrene	5.90E-04	5.96E-04	2.16E-04	1.00E-03
Fluoranthene	1.70E-03	1.44E-03	1.40E-03	1.00E-02
Pyrene	1.82E-02	1.66E-02	6.00E-05	1.00E-01
Benzo(k) fluoranthene	No Data	No Data	9.16E-03	1.00E-01
Benzo(a) pyrene	1.33E-01	1.55E-01	5.84E-02	1.00E+00
Dibenzo(a,h) anthracene	2.61E-01	1.12E-01	3.52E-01	1.00E+00
Benzo(g,h,i) perylene	3.69E-03	No Data	5.54E-03	1.00E-02
∑TEQ	4.27E-01	2.86E-01	4.28E-01	

**Table 8.** Heavy metal (HMs) mean concentration of water samples

	Station A	Station B	Station C
Arsenic (As)	0.000	0.000	0.000
Cadmium (Cd)	0.002	0.000	0.000
Chromium (Cr)	0.225	0.182	0.000
Copper (Cu)	0.287	0.007	0.018
Lead (Pb)	0.009	0.015	0.012
Nickel (Ni)	0.002	0.015	0.101
Zinc (Zn)	0.139	0.095	0.151
⅀HMs	0.664	0.314	0.282

**Table 9.** Pearson correlation of HMs components

Heavy metal components	Cadmium	Chromium	Copper	Lead	Nickel	Zinc
Cadmium	1					
Chromium	0.65	1				
Copper	**0.99**	0.62	1			
Lead	**-0.87**	-0.18	**-0.88**	1		
Nickel	-0.60	**-0.99**	-0.57	0.12	1	
Zinc	0.31	-0.52	0.35	**-0.75**	0.57	1

**Table 10.** This is a table. Tables should be placed in the main text near to the first time they are cited.

Heavy metal components	Station A	Station B	Station C	USEPA MCL value
Arsenic (As)	0.00E+00	0.00E+00	0.00E+00	1.00E-02
Cadmium (Cd)	4.00E-01	0.00E+00	0.00E+00	5.00E-03
Chromium (Cr)	2.25E+00	1.82E+00	0.00E+00	1.00E-01
Copper (Cu)	2.21E-01	5.38E-03	1.38E-02	1.30E+00
Lead (Pb)	6.00E-01	1.00E+00	8.00E-01	1.50E-02
Nickel (Ni)	2.50E+01	1.88E+02	1.26E+03	8.00E-05
Zinc (Zn)	2.32E-02	1.58E-05	2.52E-05	6.00E+00
N	6	5	4	
PLI	6.41E-01	1.75E-01	2.66E-01	
NPI	4.15E+00	1.15E+01	3.08E+01	
PERI	6.29E+00	3.86E+01	2.53E+02	

MCL: minimum contamination level; N: number of variables; PLI: pollution load index; NPI: Nemerov pollution index; PERI: potential ecological risk indices

**Table 11.** Hazard Index and lifetime cancer risk of HMs in surface water samples

HI (a)		Adult			Children		
HMs	RfD	Station A	Station B	Station C	Station A	Station B	Station C
Arsenic	3.0E-04	No Data	No Data	No Data	No Data	No Data	No Data
Cadmium	5.0E-04	2.83E-04	No Data	No Data	4.07E-04	No Data	No Data
Chromium	3.0E-03	5.30E-02	4.29E-02	No Data	7.63E-02	6.17E-02	No Data
Copper	4.0E-02	5.07E-03	1.24E-04	3.18E-04	7.30E-03	1.78E-04	4.58E-04
Lead	3.5E-03	1.82E-03	3.03E-03	2.42E-03	2.62E-03	4.36E-03	3.49E-03
Nickel	2.0E-02	7.07E-05	5.30E-04	3.57E-03	1.02E-04	7.63E-04	5.14E-03
Zinc	3.0E-01	3.27E-04	2.24E-04	3.56E-04	4.71E-04	3.22E-04	5.12E-04
∑HMs		6.06E-02	4.68E-02	6.67E-03	8.72E-02	6.73E-02	9.59E-03
**LCR (b)**		**Adult**			**Children**		
**HMs**	**CSF**	**Station A**	**Station B**	**Station C**	**Station A**	**Station B**	**Station C**
Arsenic	1.5	No Data	No Data	No Data	No Data	No Data	No Data
Cadmium	6.3	2.54E-06	No Data	No Data	3.96E-06	No Data	No Data
Chromium	0.5	2.27E-05	1.84E-05	No Data	3.53E-05	2.86E-05	No Data
Copper	No CSF	No CSF	No CSF	No CSF	No CSF	No CSF	No CSF
Lead	8.5E-03	1.54E-08	2.57E-08	2.06E-08	2.40E-08	4.00E-08	3.20E-08
Nickel	8.4E-01	3.39E-07	2.54E-06	1.71E-05	5.27E-07	3.96E-06	2.66E-05
Zinc	No CSF	No CSF	No CSF	No CSF	No CSF	No CSF	No CSF
∑HMs		2.56E-05	2.09E-05	1.72E-05	3.98E-05	3.26E-05	2.67E-05

No Data – Analytical data unavailable; No CSF: cancer slope factor unavailable; HI: Hazard index; LCR: lifetime cancer risk
